# Correlation of Circulating Omentin-1 with Bone Mineral Density in Multiple Sclerosis: The Crosstalk between Bone and Adipose Tissue

**DOI:** 10.1371/journal.pone.0024240

**Published:** 2011-09-15

**Authors:** Majid Assadi, Hooman Salimipour, Samad Akbarzadeh, Reza Nemati, Syed Mojtaba Jafari, Afshar Bargahi, Zahra Samani, Mohammad Seyedabadi, Zahra Sanjdideh, Iraj Nabipour

**Affiliations:** 1 The Persian Gulf Nuclear Medicine Research Centre, Bushehr University of Medical Sciences, Bushehr, Iran; 2 Department of Neurology, School of Medicine, Bushehr University of Medical Sciences, Bushehr, Iran; 3 Department of Biochemistry, School of Medicine, Bushehr University of Medical Sciences, Bushehr, Iran; 4 Department of Endocrine and Metabolic Diseases, The Persian Gulf Tropical Medicine Research Centre, Bushehr University of Medical Sciences, Bushehr, Iran; 5 Department of Biochemistry, The Persian Gulf Marine Biotechnology Research Centre, Bushehr University of Medical Sciences, Bushehr, Iran; Innsbruck Medical University, Austria

## Abstract

**Background:**

Patients with multiple sclerosis (MS) are at increased risk of osteoporosis and fractures. Adipose tissue-derived adipokines may play important roles in the osteoimmunology of MS. In order to determine whether omentin-1 and vaspin may be related to bone health in MS patients, we compared circulating levels of these recently identified adipokines, between MS patients and healthy controls.

**Methods:**

A total of 35 ambulatory MS patients with relapsing-remitting courses were compared with 38 age- and sex-matched healthy controls. Bone mineral density (BMD) was determined for the lumbar spine (L2–L4) and the proximal femur using dual-energy x-ray absorptiometry. Circulating omentin-1, vaspin, osteocalcin, osteopontin, osteoprotegerin, the receptor activator of nuclear factor-κB ligand, matrix metalloproteinase 9, C-reactive protein and 25-hydroxy vitamin D levels were evaluated by highly specific enzyme-linked immunosorbent assay methods.

**Results:**

There was no significant difference between the two groups regarding bone-related cytokines, adipocytokines, and the BMD measurements of patients with MS and the healthy controls. However, in multiple regression analysis, serum omentin-1 levels were positively correlated with BMD at the femoral neck (β = 0.49, p = 0.016), total hip (β = 0.42, p = 0.035), osteopontin (β = 0.42, p = 0.030) and osteocalcin (β = 0.53, p = 0.004) in MS patients. No correlations were found between vaspin, biochemical, and BMD measures in both groups.

**Conclusions:**

Elevated omentin-1 serum levels are correlated with BMD at the femoral neck and the serum levels of osteocalcin and osteopontin in MS patients. Therefore, there is crosstalk between adipose tissue and bone in MS.

## Introduction

Adipose tissue can now be considered a true endocrine organ that secrets adipokines, which are protein factors that show a number of important systemic complex interactions and influence a large number of different organ systems [1].

For the past 15 years, clinical observations and scientific evidence have revealed that adipokines play important roles in the crosstalk between bone and energy metabolisms [2]. The leptin-dependent central control of bone mass [3] and adiponectin-induced osteoblast differentiation and increasing osteoclast formation [4] suggest that adipocyte tissue-derived factors—adipokines—affect bone remodeling. However, although the relationship of leptin, adiponectin, resistin and visfatin with chondrocyte function and the skeleton have been investigated, the role of novel adipokines (e.g., omentin-1 and vaspin) in bone biology remains to be elucidated.

Omentin-1 is a novel 34 kDa adipokine that is highly and selectively expressed in visceral adipose tissue compared with subcutaneous adipose tissue [5], [6]. Furthermore, omentin-1 enhances insulin action and Akt phosphorylation [6]; it is inversely related to obesity [7] and is downregulated by insulin and glucose [8]. An increase in this novel adipokine concentration with an increase of insulin sensitivity after weight loss was reported [9].

Vaspin (visceral adipose tissue-derived serpin), as a member of the serine protease inhibitor family, is also a novel adipocytokine with insulin-sensitizing effects [10]. Circulating levels and tissue expression of vaspin increased at the peak of obesity and insulin resistance in Otsuka Long-Evans Tokushima fatty rats, an animal model of abdominal obesity and type 2 diabetes mellitus [11].

Adipokines also influence immune responses. Indeed, strong evidence suggests that complex interactions exist among adipokines and inflammatory and autoimmune diseases. The study of leptin has shown that this adipocyte-derived hormone plays a major role in the immune response in normal and pathophysiological processes, including inflammation, rheumatic diseases, and autoimmune conditions [12]–[15]. Hence, leptin may contribute to the pathogenecity of immune-mediated disorders such as MS [16]. It has been shown that leptin-deficient mice were resistant to experimental autoimmune encephalomyelitis, an animal model of human MS [17] and that this adipokine can affect the survival and proliferation of autoreactive CD4(+) T cells in experimental autoimmune encephalomyelitis [18].

The aims of this study are: 1) to compare circulating levels of omentin and vaspin, which are recently identified adipokines, between MS patients and healthy controls; 2) to correlate these adipokine levels with bone mineral density (BMD) and bone-related mediators and cytokines in order to determine whether circulating levels of these novel cytokines may be related to bone health in MS patients.

## Materials and Methods

### Patients and controls

Thirty-five consecutive definite MS patients (mean age±SD 31.57±7.26 years: 27 women and 8 men) with relapsing-remitting courses were prospectively enrolled in the study at the neurology clinic of Bushehr University of Medical Sciences. All patients were diagnosed according to McDonald's criteria [19]. The Kurtzke Expanded Disability Status Scale (EDSS) was used to score degree of disability [20]. None of the patients had immobility, had been formerly diagnosed with any bone disease, had been administered curative medication for osteoporosis, or were in menopause.

The age and sex-matched controls (30.0±6.12 years: 30 women and 8 men) were selected from a cohort of healthy subjects recruited by the Persian Gulf nuclear medicine research center at Bushehr University of Medical Sciences for evaluation of bone health in the Persian Gulf region.

The following exclusion criteria were used for MS patients and the healthy controls: 1) the known presence of generalized bone diseases including hyperparathyroidism, hypoparathyroidism, thyroid disorders, rheumatoid arthritis, Cushing disease, steroid-induced osteoporosis, renal osteodystrophy, and other metabolic diseases; 2) a history of malignant diseases and liver diseases; 3) drug addiction; and 4) restriction to bed rest within the last 2 weeks after an illness or complete bed rest for 3 months.

All subjects completed a detailed questionnaire that requested demographic and behavioral information as well as a medical history of conditions that could influence bone mass and metabolism. Venous blood was obtained from all patients and healthy controls in a fasting state. All the sera were kept frozen at −70 C until they were used.

The study was approved by the medical-ethical committee of Bushehr University of Medical Sciences, and written informed consent was obtained from all subjects.

### Measurements

#### Physical measurements

A stadiometer was used to measure height and weight. Heavy outer garments and shoes were removed before the participants' height and weight were measured. Body mass index (BMI) was calculated.

BMD was determined for the lumbar spine (L2–L4) and proximal femur (neck) using dual-energy x-ray absorptiometry on an Osteocore II bone densitometer (Osteocore II discrepancies, the same operator tested all the women during the study. Duplicate measurements were obtained from 30 women who agreed to undergo a repeat assessment on the same day, and the precision errors were calculated using the root mean square method. The coefficients of variation (CVs; precision) of measurements of the lumbar spine and femoral neck were 0.8% and 1.6%, respectively.

#### Laboratory measurements

Measurement of C-reactive protein by a CRP high sensitive enzyme-linked immunosorbent assay (hs-CRP ELISA; IBL International, GmbH, Hamburg, Germany) was conducted. The detectable concentration of the CRP HS ELISA assay was estimated to be 0.02 µg/ml. The mean intraassay and interassay CVs of the CRP assay were 4.1% to 6% and 5.8% to 6.3%, respectively.

An enzyme immunoassay (EIA) kit (Immundiagnostik, Bensheim and Biomedica, Wien) was used for measurement of 25-hydroxy vitamin D (25-OH vitamin D). The detection limit of the assay was 5.6 nmol/l. The mean intraassay and interassay CVs of the 25-OH vitamin D assay were 10.7% and 11.8% to 13.2%, respectively.

Serum OPG levels were measured using an ELISA commercial kit (Biomedica Gruppe, Vienna, Austria). The detection limit of the assay was 0.14 pmol/l. The mean intraassay and interassay CVs of the OPG assay were 4% to 10% and 7% to 8%, respectively.

RANKL levels were measured using an ELISA kit with an additional enhancement system (ampli-sRANKL; Biomedica Gruppe). The detection limit of the assay was 0.4 pg/mL. The mean intra-assay and interassay CVs of the RANKL assay were 8% to 9% and 6% to 3%, respectively.

The N-MID Osteocalcin ELISA (Nordic Bioscience Diagnostics A/S) was used for the quantitative measurement of osteocalcin in sera. The intra-assay CVs for the low (7.0 ng/mL), medium (21.8 ng/mL), and high (43.2 ng/mL) values were 3.4%, 2.0%, and 2.4%, respectively.

For the detection of osteopontin and matrix metalloproteinase 9 (MMP-9) in samples, commercially available ELISA (Quantikine, R&D Systems, Minneapolis, USA) kits were used according to the manufacturer's instructions. The mean minimum detectable dose of osteopontin was 0.011 ng/ml, with an intra-assay coefficient of variation of 4.0% and an interassay coefficient of variation of 6.6%. The mean minimum detectable dose of MMP-9 was 0.013 ng/ml, with an intra-assay coefficient of variation of 4.1% and an interassay coefficient of variation of 7.6%.

To detect vaspin in the serum samples, commercially (Cat. No. Cat. No. V0712EK) available enzyme-linked immunosorbent assay kits (AdipoGen, Seoul, Korea) were used according to the manufacturer's instructions. The assay sensitivity for vaspin was 0.012 ng/ml; the intra- and interassay coefficients of variance were 1.3% to 3.8% and 3.3% to 9.1%, respectively.

Serum omentin-1 concentrations were measured using manual omentin-1 (human) detection (ELISA kit [intelectin-1 (human) ELISA kit, Apotech Corporation, Switzerland]). The detection limit of the assay was 0.4 ng/ml (range 0.5 to 32 ng/ml). The mean intraassay and interassay CVs of the omentin-1 assay were 4.51% to 7.4% and 4.19% to 9.27%, respectively. The antibodies used in this kit are specific to measurement of natural and recombinant human omentin-1.

### Statistical Analysis

Normal distribution of the data was controlled with the Kolmogorov-Smirnov test. Probability values <5% were considered statistically significant. The significance of the difference in the results between the two groups was determined with chi-square analysis using 2×2 contingency tables. A two-tailed t-test was used to compare the values across groups in the presence of a normal distribution. Significant differences were assessed with the Mann-Whitney U test in the absence of a normal distribution.

Multiple linear regression models were used to assess the association between omentin-1 (independent variable) and BMD at a number of skeletal sites as well as bone-related biochemical parameters and cytokines (each factor was considered as a separate dependent variable in a series of models).The models were adjusted for age, BMI and serum 25-hydroxy vitamin D levels. Because the distributions of serum 25-hydroxy vitamin D, hs-CRP, RANKL, osteocalcin, osteopontin and adipocytokines were skewed, logarithmically transformed values were used for statistical analysis. The geometric mean (standard deviation) for those biochemical variables was provided.

Spearman correlation analysis was employed to study the relationships among osteopontin, osteocalcin and T scores of femoral neck.

All statistical analyses were performed using the PASW Statistics GradPack 18 (SPSS Inc., Chicago, IL).

## Results

The characteristics of the study participants are shown in Table 1. There were no significant differences in age, gender, weight, height, and BMI between the patients and the controls. Of the total of 73 participants, 2 (5.7%) and 3 (7.9%) subjects were smokers in the patient and control groups, respectively (p>0.05).

**Table 1:** Demographic, anthropometric, biochemical and bone mineral density characteristics of MS patients and healthy controls.

	MS patients	Controls	P value
Age (years)	31.57 (7.26)	30.0 (6.12)	0.320
Gender (female/male)	27/8	30/8	0.852
Weight (kg)	61.08 (11.53)	66.0 (10.91)	0.066
Height (cm)	161.68 (8.83)	162.28 (7.98)	0.760
BMI (kg/m^2^)	23.36 (4.03)	24.98 (3.19)	0.060
25-OH vitamin D (nmol/l)	32.35 (3.09)*	23.98 (2.13)*	0.087
hs-CRP (µg/ml)	1.11 (2.94)*	1.12 (3.01)*	0.286
RANKL (pg/ml)	2.07 (1.41)*	2.10 (1.59)*	0.815
OPG (pmol/l)	1.96 (0.90)	2.32 (0.90)	0.095
Osteopontin (ng/ml)	41.2 0 (2.35)*	37.67 (2.46)*	0.583
Osteocalcin (ng/ml)	1.62 (1.62)*	1.98 (1.88)*	0.446
MMP-9 (ng/ml)	20.65 (6.99)*	21.67 (3.59)*	0.413
Omentin-1 (ng/ml)	204.17 (2.01)*	191.86 (2.07)*	0.965
Vaspin (ng/ml)	1.58 (1.86)*	1.63 (1.94)*	0.774
Lumbar BMD (g/cm2)	0.964 (0.167)	1.028 (0.131)	0.072
Femur neck BMD (g/cm^2^)	1.038 (0.196)	1.00 (0.102)	0.350
Total hip BMD (g/cm^2^)	2.775 (0.573)	2.607 (0.424)	0.156

Values are means (standard deviation).

*Values are geometric means (standard deviation).

Abbreviations: BMI, body mass index; 25-OH vitamin D, 25-hydroxy vitamin D; hs-CRP, high sensitive C-reactive protein; RANKL, the receptor activator of nuclear factor-κB ligand; OPG, osteoprotegerin; MMP-9, matrix metalloproteinase 9; BMD, bone mineral density.

The median MS duration was 48.0 months (interquartile range, 12–84 months). The average EDSS score was 0.62±0.49 (13 [37.1%] patients = 0, 22 [62.9%] patients = 1). All patients had been administered pulse steroids at least once during the duration of the disease. A total of 34 patients had been administered immunomodulatory drugs (avonex = 13, sinovex = 9, rebif = 7, betaseron = 5 subjects). Four (11.4%), two (5.7%), and eighteen (51.4%) patients were on anticonvulsants, oral hypoglycaemic agents, and vitamin D supplementation, respectively.

There were no statistically significant differences in serum levels of 25-OH vitamin D levels between anticonvulsant drugs users and non-users (median = 24.68 nmol/l versus median = 32.08 nmol/l, p = 0.196). Also, there were no statistically significant differences in serum levels of 25-OH vitamin D levels between the MS patients with vitamin D supplementation and the untreated patients (median = 37.52 nmol/l versus median = 21.84 nmol/l, p = 0.506).

The serum levels of bone-related cytokines, adipocytokines, MMP-9, 25-OH vitamin D, hs-CRP, and the BMD measurements of patients with MS and the healthy controls are also shown in Table 1. There was no significant difference between the two groups regarding hs-CRP, MMP-9, osteopontin, osteocalcin, OPG, RANKL, and vaspin. There also was no significant difference in the BMD measures between the two groups. Although patients with MS had higher serum levels of omentin-1 than the control group (Table 1), this difference was not statistically significant.

BMI had a significant correlation with lumbar spine BMD (r = 0.49, p<0.0001), however, femoral neck BMD and other bone- related biochemical markers were not correlated with BMI.

Table 2 shows the correlations for serum omentin-1 in relation to age, BMI, biochemical, and BMD measures in MS patients and controls. The serum omentin-1 levels were positively correlated with osteopontin (β = 0.48, p = 0.012), osteocalcin (β = 0.50, p = 0.006) and BMD at the femoral neck (β = 0.45, p = 0.011) and total hip (β = 0.43, p = 0.015) in MS patients. In multiple regression analysis, these correlations remained significant in MS patients after adjustment for age, BMI and serum 25-OH vitamin D levels (Table 2). Moreover, omentin-1 levels were positively correlated with osteocalcin in MS patients and controls.

**Table 2:** Multiple linear regression analysis for the association between omentin-1 (independent variable), and bone mineral density, anthropometric measurements and biochemical parameters (dependent variables) in MS patients and healthy controls.

	MS patients				Controls			
	Unadjusted		Adjusted*		Unadjusted		Adjusted*	
	β	P value	β	P value	β	P value	β	P value
Age	−0.37	**0.044**	−0.35	0.060	−0.14	0.380	−0.14	0.409
BMI	−0.03	0.871	0.01	0.946	−0.01	0.964	0.02	0.880
25-OH vitamin D	0.03	0.851	0.12	0.552	−0.05	0.730	−0.04	0.816
hs-CRP	−0.01	0.978	0.08	0.655	0.08	0.630	0.04	0.810
OPG	−0.23	0.222	−0.20	0.332	−0.09	0.598	−0.05	0.746
RANKL	0.10	0.572	0.08	0.697	−0.04	0.772	−0.05	0.760
MMP-9	0.01	0.978	0.04	0.813	−0.26	0.116	−0.27	0.110
Vaspin	−0.03	0.863	−0.06	0.768	0.04	0.801	0.03	0.859
Osteocalcin	0.50	**0.006**	0.53	**0.004**	0.37	**0.021**	0.35	0.036
Osteopontin	0.48	**0.012**	0.42	**0.030**	−0.05	0.747	−0.01	0.541
Lumbar BMD	0.01	0.791	0.01	0.965	0.15	0.360	0.14	0.382
Femur neck BMD	0.45	**0.011**	0.49	**0.016**	0.23	0.154	0.21	0.207
Total hip BMD	0.43	**0.015**	0.42	**0.035**	0.18	0.264	0.18	0.286

*Adjusted for age, BMI, and serum 25-OH vitamin D levels.

Abbreviations: BMI, body mass index; 25-OH vitamin D, 25-hydroxy vitamin D; hs-CRP, high sensitive C-reactive protein; RANKL, the receptor activator of nuclear factor-κB ligand; OPG, osteoprotegerin; MMP-9, matrix metalloproteinase 9; BMD, bone mineral density.

In multiple regression analysis, after adjustments, serum omentin-1 showed no significant correlation with age and BMI in MS patients and controls (Table 2).

No correlations were found between vaspin and age, BMI, biochemical, and BMD measures in both groups (data not shown).

Serum levels of osteocalcin were significantly and positively correlated with osteopontin levels in MS patients (r = 0.61, p<0.0001) and the controls (r = 0.44, p = 0.005). Both osteocalcin (r = 0.42, p = 0.019) and osteopontin (r = 0.49, p = 0.008) were correlated with T scores of femoral neck in patients with MS.

## Discussion

Previous studies have described various adipo(cyto)kines that are implicated in bone and adipose tissue crosstalk [3], [4], [21], [22]. For example, osteoclastogenesis is increased by adiponectin via the RANKL/OPG mechanism, [4], [21] and leptin is involved in the control of bone mass by a complex mechanism that has an important role in the central nervous system and osteoblastic β2-adrenergic receptors [22]. However, there is a lack of studies on the possible effects of omentin-1 on bone metabolism in the medical literature.

In the current study, although no association could be found between omentin-1 and BMD at multiple skeletal sites in the healthy control group, we found, for the first time, that higher circulating omentin-1is associated with higher BMD at the femoral neck in patients with MS. As several lines of evidence indicate that MS is an autoimmune disorder [23], and a major role for adipocytokines in the immune system and autoimmune disorders have been suggested [14], it could be hypothesized that complex mechanisms might be involved in autoimmunity, omentin-1, and bone metabolism in MS patients.

We found a significant correlation between omentin-1 and osteopontin. Thus, osteopontin may be a possible candidate biomarker in the crosstalk between bone and adipose tissue. This pro-inflammatory cytokine is a multifunctional phosphorylated acidic glycoprotein containing an arginine-glycine-aspartate integrin binding motif that has been implicated in cell-mediated immunity, inflammation, tissue repair, and cell survival [24], [25]. Osteopontin may be produced by immune and non-immune cells including bone cells [24], [26]. The osteopontin level has been found to be the most prominent expressed cytokine within MS lesions, and increased plasma levels were reported in active relapsing-remitting MS patients [27]. In a longitudinal study, OPN levels were elevated prior to increased disease activity in relapsing-remitting MS patients [28].

This cytokine and adhesion protein is essential for stress-induced bone remodeling and bone strength because it attaches bone cells to bone matrix and generates intracellular signals for normal osteoclast motility on bone [24], [29]. Recently, increased serum osteopontin has been suggested as a biomarker for the early diagnosis of osteoporosis in postmenopausal women. [29]. In another recent study, increased osteopontin plasma levels in MS patients correlated with the bone-specific degradation product C-telopeptide of type-I collagen [30]. However, Altintas et al. [31], by introducing osteopontin as a shared cytokine in the pathogenesis of MS and osteoporosis, reported lower levels of circulating osteopontin in MS patients with osteoporosis at the femoral neck. Similar to the latter study, we also found that serum osteopontin levels were positively correlated with T-scores at the femoral neck.

Osteocalcin, an osteoblast-produced protein, is an important mediator in the reciprocal relationship between bone metabolism and adipocytes in glucose/fat metabolism. It has been associated with measures of insulin resistance, the presence of metabolic syndrome, and adipokine levels [32]. Osteocalcin can increase beta-cell proliferation and insulin secretion as well as induce adiponectin, an insulin-sensitizing adipokine, in adipocytes [2], [33]. Interestingly, in the current study, osteocalcin levels were positively correlated with omentin-1, osteopontin levels, and BMD at the femoral neck in MS patients. Therefore, it seems that there are complex mechanisms acting on BMD, osteopontin, osteocalcin, and omentin-1 in the cross-talk between bone and adipose tissue in MS patients. In fact, further studies are warranted in order to elucidate the integrative mechanisms, which contribute in bone, fat, and immunity interactions.

Matrix metalloproteinases (MMPs) are involved in the fragmentation of myelin basic protein and demyeliation in MS [34], and MMP-9 serum levels are increased in relapses [35], [36]. Because elevated serum levels of hs-CRP are associated with MS relapses, hs-CRP may be a useful surrogate marker for detection of subclinical inflammatory activity in MS [37]. We found no relationship among MMP-9, hs-CRP, and omentin-1 in patients with MS.

The relative concentration of RANKL and OPG in bone is a major determinant of bone health [38]. The RANK/RANKL/OPG system has also been shown to modulate dendritic cells and activate T cells as well as to promote B-cell maturation and antibody response, which suggests that this system plays an important role in both skeletal and immune systems [39]. This osteoimmunological system configures a wide range of molecular and cellular interactions [40]. Kurban, et al., in a small study, reported that RANKL and OPG levels were significantly higher in MS patients compared with the healthy controls [41]. Thus, they hypothesized that the RANK/RANKL/OPG system might be involved in the immune induced mechanism of osteoclastogenesis and bone loss in MS [41]. However, in the current study, there were no significant differences in RANKL and OPG serum levels between MS patients and the controls. In addition, we found no correlation among RANKL, OPG, and omentin-1 in MS patients.

It is interesting to note that serum levels of vaspin, a member of the serine protease inhibitor family and an adipokine with insulin-sensitizing effects, showed no correlations with BMD at different skeletal sites, omentin-1, osteopontin, and osteocalcin in MS patients. These results suggest that vaspin may not be involved in the cross-talk between bone and adipose tissue in MS. However, although there is a lack of studies on vaspin serum levels in relation to bone metabolism including BMD, our findings also suggest that different adipokines exert different influences on bone.

Patients with MS are at increased risk of osteoporosis and fractures because they often have multiple risk factors for osteoporotic fractures [42], reduced bone mass, and vitamin D deficiency [43]. Low BMD is prevalent in patients with MS and is related to the level of ambulatory function and physical activity [44]–[46]. Overall, evidence exists that there are correlations between EDSS with BMD at different skeletal sites [45]–[47]. Since all patients in our study were ambulatory with low EDSS scores, we did not find significant differences in BMD at different skeletal sites between the patients with MS and the healthy controls.

There are several limitations to this study. First, the sample size was small, which may have affected our statistical power. Second, we did not include patients with high EDSS scores. All patients were ambulatory and relatively young. Thus the results of the study may not be generalized to nonambulatory individuals with MS. However, evaluation of MS patients with low EDSS provided us with a relatively good milieu to investigate early pathophysiological changes in the osteoimmunology and adipose tissue. Third, causality between omentin-1 and BMD could not be clarified in our cross-sectional study. Since we assessed the investigated adipocytokines with single measurements, the changes in these adipocytokines over time could not be reflected in the current study. To clarify circulating adipocytokines in relation to worsening functional capacity and the duration of the disease, longitudinal studies are warranted. The measurement of additional adipocytokines and inflammatory markers and cytokines merits consideration in order to elucidate the complex system that regulates bone, fat, the immune system, and inflammation in MS.

A growing body of evidence supports that circulating vitamin D levels may be involved in the regulation of the clinical disease activity of MS and could be partially responsible for low bone mineral density and the progression to osteoporosis in these patients [44], [48]. Low circulating vitamin D levels were reported in MS patients [44].We also found low serum 25-OH vitamin D levels among MS patients. However, more than half of those patients were on vitamin D supplementation. It should be noted that the control subjects in the current study also had low serum 25-OH vitamin D levels. In a multi-centre study in Iran, we reported a high prevalence of moderate to severe vitamin D deficiency among different urban areas [49].

Although circulating omentin-1 levels in relation to markers of bone metabolism were adjusted for 25-OH vitamin D levels in multiple regression models in MS patients and the controls, the observed differences on correlation of bone markers/ omentin-1 between the two groups may be attributable rather to different distribution of 25-OH vitamin D-status than the cohorts being MS patients or controls per se.

In conclusion, elevated omentin-1 serum levels are correlated with BMD at the femoral neck, and serum levels of osteocalcin and osteopontin in MS patients. However, no correlations could be found for vaspin, as an adipokine, in relation to BMD and bone metabolism. The observed omentin-1 in relation to bone metabolism in MS, as an autoimmune disorder, supports the recent notion that adipose tissue has close cross-talk with bone and the immune system as well as sharing an abundance of molecules and regulatory mediators in complex mechanisms. The clarification of this intermingled bone and adipocytokines complex system in the osteoimmunology spectrum by integrative pathophysiological approaches might promise attractive and novel pharmacological insights for treatment of both osteoporosis and autoimmune disorders such as MS in the future.
